# Fetal Membrane Epigenetics

**DOI:** 10.3389/fphys.2020.588539

**Published:** 2020-12-03

**Authors:** Tamas Zakar, Jonathan W. Paul

**Affiliations:** ^1^Department of Maternity & Gynaecology, John Hunter Hospital, New Lambton Heights, NSW, Australia; ^2^School of Medicine and Public Health, Faculty of Health and Medicine, The University of Newcastle, Callaghan, NSW, Australia; ^3^Priority Research Centre for Reproductive Science, The University of Newcastle, Callaghan, NSW, Australia; ^4^Hunter Medical Research Institute, New Lambton Heights, NSW, Australia

**Keywords:** amnion, chorion, decidua, chromatin modifications, non-coding RNAs, human pregnancy, parturition

## Abstract

The characteristics of fetal membrane cells and their phenotypic adaptations to support pregnancy or promote parturition are defined by global patterns of gene expression controlled by chromatin structure. Heritable epigenetic chromatin modifications that include DNA methylation and covalent histone modifications establish chromatin regions permissive or exclusive of regulatory interactions defining the cell-specific scope and potential of gene activity. Non-coding RNAs acting at the transcriptional and post-transcriptional levels complement the system by robustly stabilizing gene expression patterns and contributing to ordered phenotype transitions. Here we review currently available information about epigenetic gene regulation in the amnion and the chorion laeve. In addition, we provide an overview of epigenetic phenomena in the decidua, which is the maternal tissue fused to the chorion membrane forming the anatomical and functional unit called choriodecidua. The relationship of gene expression with DNA (CpG) methylation, histone acetylation and methylation, micro RNAs, long non-coding RNAs and chromatin accessibility is discussed in the context of normal pregnancy, parturition and pregnancy complications. Data generated using clinical samples and cell culture models strongly suggests that epigenetic events are associated with the phenotypic transitions of fetal membrane cells during the establishment, maintenance and termination of pregnancy potentially driving and consolidating the changes as pregnancy progresses. Disease conditions and environmental factors may produce epigenetic footprints that indicate exposures and mediate adverse pregnancy outcomes. Although knowledge is expanding rapidly, fetal membrane epigenetics is still in an early stage of development necessitating further research to realize its remarkable basic and translational potential.

## Introduction

Current consensus defines an epigenetic trait as “a stably heritable phenotype resulting from changes in a chromosome without alterations in the DNA sequence.” (Berger et al., 2009) The fetal membranes and the adjacent decidua adopt a phenotype in early gestation that supports pregnancy by preserving the integrity of the gestational sac, reducing myometrial contractility and controlling maternal innate and adaptive immunity to tolerate the semi-allogenic fetus. These protective characteristics are maintained stably throughout pregnancy despite the massive growth of the gestational sac. At term, a phenotype transition occurs that promotes membrane rupture, myometrial contractions, inflammation and lowered immune tolerance (Menon et al., 2016, 2019). The changes trigger birth. Similar changes can be elicited by pathological conditions, such as genital tract infection, pre-eclampsia and uterine overdistension, often inducing birth before term (Romero et al., 2014).

Epigenetic events establish, sustain and adjust chromatin structure in a cell-specific fashion through DNA methylation, post-translational histone modifications and regulatory non-coding RNAs. The resulting chromatin landscapes determine cell-specific gene expression patterns, which determine tissue phenotypes (Roadmap Epigenomics Consortium et al., 2015). Evidence is accumulating that characteristic chromatin modification patterns and non-coding RNA transcriptomes occur in the fetal membrane and decidua cells and change dynamically during normal and pathological pregnancies. This suggests that the mechanisms driving the phenotype transformations during gestation and at birth are, at least partially, epigenetic. This article summarizes information about epigenetic processes and associated phenotype changes in the amnion, chorion laeve and the decidua and consider their significance in normal pregnancies, during labor and in pregnancy disorders.

## Amnion

In primates, amnion epithelial cells differentiate from epiblasts at about Day 8 of pregnancy, which is prior to gastrulation (Enders et al., 1986; Sasaki et al., 2016). The pluripotent epiblast phenotype is preserved throughout pregnancy to a substantial degree, as evidenced by the ability of amnion cells to differentiate to cells of all three germ layers *in vitro* under appropriate conditions (Easley et al., 2012). During the most of the gestation, the amnion exhibits characteristics that are anti-inflammatory and smooth muscle relaxant in agreement with a role in protecting the pregnancy (Carvajal et al., 2006; Silini et al., 2013). Furthermore, the mechanical strength of the gestational sac is provided by the strong collagenous (“compact”) layer of the amnion membrane, which is maintained by the fibroblastic (mesenchymal) cells of the connective tissue underlying the epithelial layer. There is now evidence that the mesenchymal cells may be derived from the epithelial cells that undergo reversible epithelial-to-mesenchymal cell transformation (EMT) allowed by their phenotypic plasticity. There is also evidence that EMT in the amnion occurs increasingly with advancing gestation and in response to the proinflammatory cytokine, TNFα, or oxidative stress, which intensifies remodeling and results in the mechanical weakening of the membrane (Janzen et al., 2017; Richardson et al., 2020). Moreover, amnion mesenchymal cells respond strongly to proinflammatory stimuli, further promoting membrane rupture and the production of uterotonic factors including prostaglandins (Whittle et al., 2000; Sato et al., 2016). Thus, controlled and properly timed phenotype transitions of the amnion cells are critical for both maintaining pregnancy and triggering birth. Epigenetic events impacting on gene expression patterns are believed to contribute to the gestational transformations of amnion cells, which is stimulating interest in the topic.

### DNA Methylation

Methylation of cytosines at the 5th position in the CpG motifs of DNA (5meCpG) is the most thoroughly studied epigenetic chromatin modification. CpG methylation is traditionally considered to silence genes by promoting closed chromatin structure or recruitment of repressor complexes to gene regulatory regions (Jones, 2012). There are about 28 million CpG sites in the human genome and more than 80% of these are methylated (Breiling and Lyko, 2015; Lövkvist et al., 2016). The density of DNA methylation in particular chromatin regions depends on the frequency of CpG dinucleotides, which varies substantially in the genome. Recent global analyses of CpG methylation levels have indicated, however, that the relationship of DNA methylation to gene activity depends on the genomic context. For example, higher CpG frequency regions, called CpG islands (Gardiner-Garden and Frommer, 1987), are generally unmethylated in promoters, and CpG sites are highly methylated in the transcribed regions of active genes. Low CpG density promoters and regulatory regions may exhibit variable methylation associated with variable gene activity, which is often tissue-specific and change with cellular differentiation (Jones, 2012).

Although DNA CpG methylation is considered a stable and mitotically heritable epigenetic modification, it undergoes turnover catalyzed by DNA methylating and demethylating enzyme systems (Schubeler, 2015; Kim and Costello, 2017). DNA methylation in human cells is performed by a family of DNA methyl transferases (DNMTs), which includes DNMT1, DNMT3A, -3B, and DNMT3L. DNMT1 is generally responsible for “maintenance” methylation during the S- (DNA-replicating) phase of the cell cycle because of its selectivity toward hemi-methylated CpG motifs in the nascent double-stranded DNA. DNMT3A and -3B perform “*de novo*” methylation at unmethylated CpG sites. DNMT3L is a catalytically inactive essential cofactor of DNMT3A and -3B. The characteristics of DNA methyltransferases have been extensively studied and reviewed in the literature (Tajima et al., 2016; Gowher and Jeltsch, 2018). DNMTs (except for DNMT3L) are expressed in the amnion and the decidua (Grimaldi et al., 2012; Mitchell et al., 2013) and are discussed in later sections. A group of 5meCpG-binding proteins (MBDs) recognize and functionally interpret DNA methylation patterns as signals for gene repression in most genomic contexts (Du et al., 2015). The mechanisms include heterochromatin formation, establishment of repressive histone modifications, nucleosome remodeling and extension of methylated DNA regions. Of the 11 MBD proteins identified so far (Du et al., 2015) one, MBD5, has been reported in decidualizing endometrial cells (Grimaldi et al., 2012). MBD proteins and their roles as methylated DNA readers is a major unexplored area of fetal membrane epigenetics.

The elucidation of the biochemical mechanisms that erase the 5meCpG modification was challenging because of the large energy barrier obstructing the direct enzymatic removal of the 5-methyl group. The best characterized removal pathway is initiated by the oxidative modification of the 5-methyl group producing 5-hydroxymethyl cytosine (5-hmC). The reaction is catalyzed by the Ten-Eleven Translocation group of dioxygenases (TET-1, -2, and -3), which use molecular oxygen and 2-oxoglutarate as co-substrates. TETs can oxidize 5-hmC further to 5-formyl and 5-carboxyl cytosine, which are detected by the base excision DNA repair (BER) system eventually replacing 5meC with unmodified cytosine in the CpG motifs (Kohli and Zhang, 2013; Bochtler et al., 2017). The central role of 5-hmC in DNA demethylation is reinforced by the inefficiency of DNMT1 to recognize 5-hmCpG for maintenance methylation, which results in the “passive” loss of methylation of the affected 5meCpGs during replication (Hashimoto et al., 2012; Kohli and Zhang, 2013). Thus, 5-hmC is generated from 5meC and as a consequence its incidence is lower than that of 5meC in somatic cells (Globisch et al., 2010). Its presence, however, indicates sites of dynamic DNA methylation in the genome such as poised enhancers, low- and intermediate CpG density promoters and bivalent promoters that are subject to developmental regulation (Yu et al., 2012; Gao and Das, 2014). Finally, proteins that bind 5-hmCpGs with high affinity and may function as epigenetic readers of 5-hmCpG and its oxidized derivatives have been discovered (Spruijt et al., 2013) suggesting that these modified bases may carry epigenetic information in addition to their role in 5meCpG turnover.

DNA methylation in the fetal membranes has been studied so far using traditional techniques that do not discriminate between 5meC and 5-hmC such as bisulfite conversion (Huang et al., 2010) or have no verified selectivity between the two modified bases (e.g., enzyme-based assays). These studies are mostly descriptive, and the results are reported in terms of CpG methylation. The main findings are summarized and discussed in the following sections.

#### Genome-Wide Profiling

CpG methylation has been profiled genome-wide in the amnion using the Illumina Infinium HumanMethylation27k BeadChip (HM27k) array (Eckmann-Scholz et al., 2012). The array features probes targeting over 25,000 CpG sites preferentially in promoter CpG islands with approximately 2–3 sites per gene. The probed CpGs showed bimodal distribution with either high or low levels of methylation in cells isolated from mid-trimester amniocentesis samples and expanded in culture. Such distribution is expected in somatic cells (Pidsley et al., 2016). Methylation patterns distinguished amnion-derived cells and villous chorion samples at similar gestational age as determined by principal component analysis (PCA) and hierarchical clustering (Eckmann-Scholz et al., 2012). However, amniotic fluid may contain cells from fetal skin, airways and intestine, potentially confounding the characterization of cells originating from amnion tissue. In another study (Kim et al., 2013), amnion tissue samples from women after term and preterm labor and term not in labor were processed for methylation analysis with the HM27k array. PCA analysis robustly separated the amnion samples according to the presence or absence of labor, but not according to gestational age. Nevertheless, more than 60 genes were found to be differentially methylated at term labor versus preterm labor and term not in labor versus term labor (*p* < 0.0001), with no overlap among the top 15 differentially methylated genes in the two comparisons. Yoo et al. (2018) used the more advanced HM450 BeadChip (HM450k) containing over 485,000 probes offering a much wider genomic coverage that includes CpG islands, shores and shelves, gene bodies, untranslated regions and enhancers (Pidsley et al., 2016). Amnion tissues after term and preterm delivery were compared and methylation differences were matched to differential gene expression determined by whole transcriptome sequencing. Nearly 36,000 differentially methylated CpG sites and over 1,000 differentially expressed genes were found, of which, 71 genes exhibited reciprocal changes of expression and CpG methylation in either direction. Two genes related to cell adhesion, integrin subunit alpha 11 (*ITGA11*) and trombospondin-2 (*THBS2*), were selected for verification using bisulfite pyrosequencing and real-time RT-PCR with independently collected samples. Both genes showed lower methylation and higher expression preterm than at term. Although this study used genome-wide discovery approaches to find previously discovered and expected relationships between gene methylation and expression, it has confirmed that CpG methylation in the amnion is dynamic and related to transcriptional activity at a subset of genes linked to tissue function.

Pre-eclampsia is a severe pregnancy complication characterized by hypertension, proteinuria and maternal inflammatory reactions. Its pathogenesis is unclear but abnormal placentation, angiogenic imbalance and endothelial dysfunction are well-documented attributes of the condition. DNA methylation has been studied in the early onset form (<34 weeks of gestation) of the disease using formaldehyde-fixed, paraffin-embedded full thickness fetal membrane samples that included amnion, chorion and attached decidua (Ching et al., 2014). CpG methylation profiling with the HM450k system showed nearly 10,000 differentially methylated CpG sites with mostly increased methylation in pre-eclampsia. Differentially methylated CpGs in gene-annotated genomic regions revealed decreased methylation in promoters and increased methylation within gene bodies, consistent with widespread transcriptional activation. A number of these genes and associated pathways have been found previously to be activated in pre-eclamptic placentae. Promoter hypomethylation has also been found in several pri-microRNA (pri-miRNA) genes, indicating the epigenetic regulation of miRNA expression. Thorough bioinformatic analysis has established the fetal membranes/decidua as epigenetic responders to the pre-eclamptic condition, but tissue-specific responses remained uncharacterized because of the use of unseparated full thickness membrane samples. Suzuki et al. (2016) addressed cellular heterogeneity in the amnion by performing whole genome bisulfite sequencing with separated amnion epithelial and mesenchymal cells. They found cell-specific methylation patterns and identified one CpG site (in an intron of the *SIPA1L1* gene) with a robust cell type-specific methylation difference, which could be used as a marker to correct for the variable cell type composition in amnion tissue samples. Genome wide methylation analysis was conducted with an assay called HELP-tagging, which utilized the methylation sensitive restriction enzyme, *Hpa*II. With this assay the authors surveyed the methylation state of over 545,000 CpG sites in normal versus pre-eclamptic amnion samples (62, overall) and found 4,058 differentially methylated sites in 3,035 genes. Methylation of 123, 85, and 99 sites were influenced by systolic blood pressure, proteinuria grade and the combination of the two, respectively, in regression models. RNAseq with a subset of samples revealed that 41 genes were differentially expressed in pre-eclampsia; however, none of the differentially methylated sites were in the vicinity of the differentially expressed genes, indicating the complexity of 5meCpG-mediated gene regulation. Overall, the presence of pre-eclampsia-associated epigenetic “signatures” in the amnion is remarkable, because this tissue is not a prime player in the disease. It appears, however, that epigenetic plasticity makes the amnion a useful surrogate to report the effects of adverse intrauterine conditions on fetal tissues in general, which may impact on disease susceptibility later in life.

#### Candidate Genes

Methylation analysis of candidate genes having well-established functions is a straightforward approach to explore the role of DNA methylation in gene regulation with physiological relevance. Wang et al. (2008) examined the methylation of the *MMP1* gene promoter in the amnion in normal pregnancies and in cases of preterm pre-labor rupture of the membranes (pPROM). Matrix metalloproteases (MMPs), such as MMP1, have a key role in breaking down the collagen matrix of the amnion contributing to membrane rupture at term and preterm birth. The *MMP1* proximal promoter contains no CpG island, only 14 sporadic CpG sites, which are fairly highly methylated (≈50%) in amnion samples, as determined by clonal bisulfite sequencing. Treatment of primary cultures of amnion mesenchymal cells with the DNA-demethylating agent, 5-aza-3′-deoxycytidine (5-AZA), increased *MMP1* expression and decreased the methylation of one CpG motif at 1,538 bases upstream of the transcription initiation site. Interestingly, the methylation level of this site was significantly lower in amnion samples from deliveries complicated by pPROM compared to normal term controls. This group also described a previously undetected polymorphism in the *MMP1* promoter that generated a new CpG site as a minor allele. The presence of this CpG reduced *MMP1* promoter activity in amnion mesenchymal cells and even more so when it was methylated. *In vivo*, this site was always methylated and, remarkably, was significantly protective of pPROM in an African American population. This study illustrates that epigenetic and genetic differences may combine forming complex patterns of regulation that can be understood by analyses involving both levels. In the case of *MMP1*, the effect of DNA methylation on gene activity interacted with the polymorphism of the promoter shaping the impact on fetal membrane integrity and the risk of pPROM.

TIMP1 is a protease inhibitor protein that interacts with MMPs to control extracellular matrix remodeling. The *TIMP1* proximal promoter contains no CpG island and the 25 CpG sites around the transcriptional start site (−275)–(+279) are highly methylated in the amnion of female fetuses compared to males, as shown by Vincent et al. (2015), using the Sequenom Epityper technology. This is not surprising, since the gene is located in the X chromosome and one copy is in the hypermethylated homologous X chromosome in females. Notably, however, *TIMP1* mRNA abundance was higher in female than in male amnions and mRNA expression did not change with spontaneous labor despite a significant decrease of promoter methylation. Lipopolysaccharide (LPS) treatment of amnion explants increased methylation without effect on expression. Pre-treatment with the DNA demethylating agent, 5-AZA (5 uM, 48 h), did not affect *TIMP1* mRNA, or *TIMP1* promoter methylation levels in male or female amnions but sensitized the tissues to respond to LPS with increased *TIMP1* expression. These data suggest that *TIMP1* promoter methylation is dynamic but not linked to the level of gene activity in the amnion. Responsiveness to LPS might be influenced by methylation possibly at remote sites *in trans*.

Methylation of candidate genes in the amnion has been assessed in two further studies using the Methyl-Profiler PCR system (Mitchell et al., 2013; Sykes et al., 2015). The Methyl-Profiler (or MethylScreen) technology employs methylation-dependent and methylation sensitive restriction enzymes to probe the methylation density of pre-selected DNA sequences (Holemon et al., 2007). Labor-associated inflammatory genes (*PTGS2*, *BMP2*, *NAMPT/PBEF*, *CXCL2*), steroid receptor genes (*ESR1*, *PGR*, *NR3C1/GR*) and renin-angiotensin system components (*ACE*, *ATP6AP2/PRR*, *AGTR1*, *CTSD*, *KLK1*) were examined with the technique for promoter methylation density in amnion samples from early gestation (11–17 weeks) and after term delivery with or without labor. With the exception of *KLK1* (kallikrein 1), the proximal promoters of these genes have relatively high CpG density overlapping with CpG islands. Methylation densities of these promoters showed bimodal distribution with either highly methylated or sparsely methylated copies. The distributions did not change with gestational age or with labor but varied between genes and among individuals. Furthermore, the distribution of highly versus sparsely methylated promoter copies did not correlate with expression levels but correlated significantly between individuals. This suggests that the methylation of these promoters was established in early pregnancy (before 11–17 weeks) in a gene-specific fashion under the influence of individual conditions and was maintained until after delivery. In agreement with this, the expression of the DNMTs, DNMT1 and -3a, were highest in early pregnancy and decreased by term. Interestingly, the low CpG *KLK1* promoter exhibited intermediate methylation density in some of the samples and a loss of methylation at term, indicating dynamic methylation without a significant influence on expression level.

#### Histone Modifications

Covalent post-translational modifications of histones, which include acetylation, methylation, phosphorylation, ubiquitination, and sumoylation, are pervasive throughout the chromatin and are organized in patterns characteristic of genomic features, such as promoters, enhancers, transcribed sequences and heterochromatin regions (Roadmap Epigenomics Consortium et al., 2015). They contribute to functional states such as open or closed chromatin, active or repressed genes, poised or operative enhancers. Recent evidence also indicates that modified histones are involved in directing DNA methyltransferases to chromatin regions where DNA methylation occurs during replication and cell differentiation (Fu et al., 2020). For example, DNMT3A and -3B contain PWWP domains that bind trimethylated lysine-36 in histone 3 (H3K36me3) (Rona et al., 2016), potentially directing these enzymes to exonic sequences of transcribed genes that are highly methylated and rich in H3K36me3. In addition, targeting of DNA methylation to heterochromatic regions through interactions between DNMT3A/B and methylated H3K9, or the lysine methyltransferase establishing this gene silencing modification (G9a), or chromodomain (methyl-lysine binding) proteins associating with methylated H3K9, has been reported but the molecular mechanisms are still unclear (Rose and Klose, 2014). Moreover, DNMT3 proteins contain ADD domains, which specifically recognize unmethylated H3K4 (H3K4me0), potentially explaining the antagonism between DNA methylation and H3K4 methylation genome wide (Fu et al., 2020). Even maintenance methylation by DNMT1 has been shown to depend on histone modifications such as H3K9 methylation and H3K27 ubiquitination [reviewed by Rose and Klose (2014)].

Despite their significance in epigenetic regulation, histone modifications are still scantly characterized in the fetal membranes. For example, genome-wide screens performed routinely using chromatin immunoprecipitation with antibodies selective for modified histones (ChIP-seq) have not been published with amnion or chorion to inform about gestational changes or pathological alterations. Studies employing ChIP combined with PCR, however, have demonstrated the presence of histone modifications in the promoter regions of a few labor-associated genes in the amnion. Gene activating histone-3 and -4 acetylation (H3ac, H4ac) and histone-3, lysine-4 demethylation (H3K4me2) have been reported at the promoter of the *PTGS2* gene, which encodes a key enzyme of prostaglandin biosynthesis (Mitchell et al., 2008, 2011). Further, H4ac levels were significantly elevated at term labor when *PTGS2* expression increased (Mitchell et al., 2008). Histone-3, lysine-4 trimethylation (H3K4me3) and histone-3, lysine-27 trimethylation (H3K27me3), which are activating and repressive chromatin marks, respectively, were assessed at the promoters of *PTGS2* and two other inflammatory genes, *NAMPT/PBEF/*visfatin and *BMP2*, in amnion tissues collected in early pregnancy (10–18 weeks) and at term (Mitchell et al., 2019). The expression of these genes increased robustly at the end of gestation. Both histone modifications were present at the promoters, and sequential double ChIP showed that the same promoter copies were marked by H3K4me3 as well as H3K27me3, indicating epigenetic “bivalence.” Remarkably, bivalence was significantly reduced at term by the loss of the repressive H3K27me3 mark, indicating a shift toward a state poised for expression. H3K4 methyl transferases and H3K27me3 demethylases were expressed increasingly in the tissues with advancing pregnancy, potentially mediating the changes in histone methylation. This study suggests that an epigenetic process activating bivalently marked genes participates in the mechanism stimulating labor at term. The concept, however, has to be corroborated by the genome-wide profiling of H3K4me3 and H3K27me3 levels at gene regulatory regions in fetal membrane cells collected at different times during gestation.

Histone deacetylases are a diverse group of chromatin-modifying enzymes comprising 18 members classified into four groups (Seto and Yoshida, 2014). They are excellent drug targets, and histone deacetylase inhibitors and activators of varying isoform selectivity can be used as tools to explore the involvement of histone acetylation in gene expression control. Using this approach, Poljak et al. (2014) determined that Class II histone deacetylases may participate in the up-regulation of matrix metalloprotease-9 (MMP9) expression by ILβ in cultured amnion cells, while Class III histone deacetylases inhibit it. Similarly, the histone deacetylase inhibitor, TSA (Trichostatin A), reduced ILβ-stimulated PTGS2 expression in amnion explants supporting a role of histone acetylation in the action of the cytokine (Mitchell, 2006). The involvement of histone acetylation in these activities still needs to be confirmed by demonstrating cognate operational changes in acetyl histone levels at the gene regulatory regions since many non-histone proteins are also acetylated and are substrates for histone deacetylases (Seto and Yoshida, 2014). Therefore, drugs interfering with protein acetylation may cause global changes in the acetyl-proteome of the cells with functional consequences not necessarily mediated by histone acetylation-dependent epigenetic events (Norris et al., 2009; Orren and Machwe, 2019). An example for this has been found in the pregnant human myometrium, where TSA treatment *ex vivo* preserves progesterone receptor expression in its non-laboring state (Ilicic et al., 2017) potentially by epigenetic mechanisms (Ke et al., 2016; Ilicic et al., 2019) and reduces contractility by an extranuclear action that increases heat shock protein 20 acetylation promoting actin depolymerization and relaxation (Karolczak-Bayatti et al., 2011).

#### Non-coding RNAs

Transcription is not restricted to chromatin regions encoding protein-coding genes, but it is widespread throughout the genome. The resulting non-coding RNAs vary in size and function (Kung et al., 2013). The long non-coding RNA class, called lncRNAs, are over 200 bases long and have been implicated in epigenetic regulation by recruiting chromatin modifying protein complexes to the DNA regions to which they are tethered by complementary sequences. A well-characterized example is Xist RNA, which directs polycomb-regulatory complex-2 (PRC2) (catalyzing H3K27me3-dependent repression) to the X chromosome during X chromosome inactivation [reviewed by Kung et al. (2013)]. Long ncRNAs have been implicated in a range of reproductive disorders via epigenetic mechanisms (Shen and Zhong, 2015). Profiling of lncRNAs in (villous) placenta using a dedicated microarray covering over 33,000 (curated) lncRNAs (Arraystar Human LncRNA Array v2.0) revealed numerous differentially expressed lncRNAs in association with term and preterm birth and pPROM in two studies (Luo et al., 2013, 2015). Ten differentially expressed natural antisense lncRNAs have been paired with differential mRNA expression from the same loci arguing for functional relationships (Luo et al., 2015). It will be important to extend these studies to the fetal membranes since integrated lncRNA, chromatin modification and gene expression profiling could reveal lncRNA-mediated epigenetic events involved in normal birth, pPROM and immune regulation in tissues covering most of the maternal-fetal interface.

A distinct group of lncRNAs, called pri-miRNAs, is processed into small, 22 nucleotides long RNA fragments, called micro-RNAs (miRNAs). Micro-RNAs act as guides to direct protein complexes to mRNAs or non-coding RNAs by sequence recognition (Hammond, 2015). Depending on the degree of complementarity, this results in (m)RNA degradation and/or the inhibition of mRNA translation to proteins. By conservative estimate, there are 2,300 human miRNAs validated to date (Alles et al., 2019). Because of the tolerant complementarity, a particular miRNA may be predicted to target numerous, possibly hundreds, of mRNA species or non-coding RNAs and a particular RNA species can concurrently interact with several miRNAs. Micro-RNAs, therefore, are proposed to have homeostatic roles providing robustness to cell phenotypes by dampening the effects of stochastic fluctuations of transcription (O’brien et al., 2018). Remarkably, the miR-200 family and the ZEB transcription factors were proposed to participate in a bi-stable double negative feedback loop that controls epithelial-mesenchymal transition in epithelial cell lines. The phenotype switch may be triggered by TGFβ and is reinforced by DNA methylation at the miR-200c∼141 promoters (Gregory et al., 2011). This interaction is just one example of the crosstalk between the micro-RNA and chromatin modification aspects of epigenetic regulation. Genes encoding micro-RNAs both at intergenic and intronic locations are subject to regulation by DNA methylation and histone modifications. The control is reciprocal, since DNMTs, TATs and histone modifying enzymes are targeted by micro-RNAs in normal and diseased (e.g., cancerous) cells (Chhabra, 2015; Yao et al., 2019). In the amnion, TGFβ-driven epithelial-mesenchymal transition occurs reversibly during gestation and apparently irreversibly at labor leading to membrane rupture (Janzen et al., 2017). Many molecular details of this process have been determined recently (Richardson et al., 2020); however, the contribution of epigenetic mechanisms, including miRNAs, is still unknown despite numerous possibilities identified in cancer cells undergoing analogous changes of phenotype (Serrano-Gomez et al., 2016). Epigenetic regulation underpinning the epithelial-mesenchymal transition of amnion cells is a promising new frontier of fetal membrane research.

##### Micro-RNAs in the Fetal Membranes

Montenegro et al. (2007) have profiled miRNAs in chorioamnionic membrane samples (with attached decidua) using the TaqMan MicroRNA qRT-PCR Assays Human Panel (Applied Biosystems–Early Access kit), which assays 157 miRNAs. Most (>150) of the tested miRNAs were detected, of which 13 had decreased levels with advancing pregnancy in women after preterm birth without histological chorioamnionitis. No differences were detected with term labor. In a subsequent study by the same group (Montenegro et al., 2009), 455 miRNAs were tested using the miRCURY LNA (Exiqon) microarray (v.8.1). Here, 39 differentially expressed miRNAs were found and most (79.5%) showed lower expression at term labor compared to preterm labor. One of them, miR-338, was verified experimentally in decidual cells to target *PLA2G4B* mRNA, which encodes a phospholipase involved in prostaglandin biosynthesis. They have also demonstrated a marked down-regulation of *Dicer*, a key enzyme of miRNA biogenesis, with advancing pregnancy in agreement with a widespread reduction of miRNA levels. This finding suggests that the homeostatic role of miRNAs, which is to stabilize a transcriptome that maintains the pregnancy-supporting phenotype of the membranes, is weakened at term, thus facilitating the transition to a labor-promoting state. An even higher number of miRNAs (875 miRNAs included in the miRCURY Array v.11 from Exiqon) have been tested in isolated amnion tissues at term and after preterm labor (Kim et al., 2011). This analysis found 32 differentially expressed miRNAs between the placental and extra-placental (reflected) regions of the amnion with 31 exhibiting lower levels in the reflected part. Moreover, down-regulation of the miR-143/miR-145 cluster has been verified by qRT-PCR in the reflected amnion at term labor and miR-143 has been shown to target *PTGS2* mRNA in a transfection assay with amnion mesenchymal cells. Collectively, the above series of studies suggests that a widespread decrease in miRNA expression plays a role in the labor-promoting proinflammatory switch in the fetal membranes at term. Post-transcriptional de-repression of genes of the prostaglandin biosynthetic pathway has been identified by targeted experiments as part of this process.

The miRNA profile of term amnion has been examined in obese women (with pre-pregnancy body-mas index >30) by Nardelli et al. (2014) using the TaqMan human MicroRNA Panel v.1.0, which contains 365 miRNAs. Seventy one percent of the tested miRNAs were detected in the amnions, of which 7 miRNAs were found only in obese women. The study also found 25 miRNAs that were differentially expressed in obese versus non-obese mothers. Further, Enquobahrie et al. (2015) explored the association of 8 preselected miRNAs with preterm birth of various clinical presentations (spontaneous, pPROM, pre-eclampsia) in the amnion and the chorion leave, based on the involvement of these miRNAs in placental pathologies (Enquobahrie et al., 2015). The two fetal membrane tissues expressed these miRNAs differentially, and miR-210 and miR-233 levels in the amnion, but not in the chorion leave, were inversely associated with preterm birth risk. These pioneering studies indicate that unfavorable conditions affect miRNA expression in the amnion, which may contribute to adverse pregnancy outcomes.

## Decidua

The decidua is the endometrium of pregnancy and, being a maternal tissue, is not part of the *fetal* membranes by strict definition. It forms the maternal side of the maternal-fetal contact zone, however, and is fused with the chorion leave so intimately that it is practically impossible to separate them completely, even with sharp dissection (Mitchell and Powell, 1984). The close contact predicts functional interactions, which warrants including an overview of the epigenetics of the human decidua in this chapter to provide context for the anatomical unit often referred to as the “choriodecidua.” We focus on decidual stromal cells acknowledging that the decidua in pregnancy contains a complex and dynamic array of leukocytes (Gomez-Lopez et al., 2010), which are also subject to epigenetic regulation (Kim et al., 2012; Walsh et al., 2017). Comprehensive reviews, including the epigenetic aspects of decidual differentiation, have been published (Guo, 2012; Gao and Das, 2014; Liu et al., 2019).

### DNA Methylation

Differentiation of the endometrium to decidua involves the transformation of endometrial stromal cells to decidual cells (Zhu et al., 2014), which has similarities to mesenchymal-to-epithelial phenotype transition (Zhang et al., 2013). It occurs in the non-pregnant uterus during the progesterone-dominated secretory phase of the menstrual cycle and its proper execution is essential for successful pregnancy. The role of DNA methylation in the process was explored initially by determining the expression of DNMTs in the endometrium during the menstrual cycle and in *in vitro* models, where decidual differentiation was induced by combined progestogen (progesterone, P4 or medroxyprogesterone acetate, MPA) and estradiol (E2) or cAMP treatments of cultured endometrial stromal cells (Yamagata et al., 2009; Vincent et al., 2011; Grimaldi et al., 2012; Logan et al., 2013). These studies showed down-regulation of DNA methyl transferase expression during decidualization. Likewise, 5-AZA (a DNA methyl transferase inhibitor) treatment fostered a phenotype in the endometrial culture system reminiscent of the decidual state (Logan et al., 2010). In spite of these observations, no change in the global level of DNA methylation has been detected (Grimaldi et al., 2012), suggesting that DNA methylation changes during decidual transformation may involve alterations of methylation pattern rather than changing the overall methylation degree. CpG site-specific methylation during decidualization *in vivo* has been investigated using the Illumina HM27k array (Houshdaran et al., 2014). The top 10% of probes reporting variable CpG methylation (2,578 probes) effectively separated samples from the proliferative and secretory phases of the cycle by unsupervised cluster analysis. Differential methylation analysis, however, identified just 66 CpGs with altered methylation, with several of them associated with genes important in endometrial biology. The follow-up study using primary endometrial fibroblasts decidualized *in vitro* by P4 and E2 indicated methylation changes at several CpGs and associated genes detected *in vivo* (Houshdaran et al., 2014). In a similar study, Maekawa et al. (2019) used the more comprehensive HM450k array and found only 23 differentially methylated CpGs after MPA + E2 treatment without change in the expression of the associated genes. Moreover, the DNA methylation status of the decidual marker genes *PRL* and *IGFBP1* showed no change after differentiation induced by MPA + E2, as determined by bisulfite (clonal) sequencing. Collectively, these data suggest that DNA methylation dynamics is slight in the decidua and the involvement of CpG methylation in the decidualization process is subtle. Other epigenetic processes, such as histone modifications, may play a more predominant role.

### Histone Modifications

Decidual transformation of endometrial stromal cells *in vivo* and *in vitro* is associated with the decreasing expression of EZH2, which is the catalytic component of the histone methyltransferase complex, PRC2 (Grimaldi et al., 2011). PRC2 methylates the lysine-27 residue of histone-3, establishing the repressive H3K27me3 modification. Reduced EZH2 activity was accompanied by lowered H3K27me3 levels at the *PRL* and *IGFBP1* genes and the increased expression of the gene products, prolactin and insulin-like growth factor-binding protein 1, which are the best characterized markers of decidualization. However, the global H3K27me3 level remained unaltered after EZH2 down-regulation, indicating the locus selectivity of the histone modification changes (Grimaldi et al., 2011). Genome-wide survey of H3K27me3-marked sites by chromatin immunoprecipitation coupled to microarray promoter analysis (ChIP-chip technology, using the NimbleGen Human ChIP-chip 3 × 720 K RefSeq promoter array) confirmed the global rearrangement of the H3K27me3 pattern. Upon decidual transformation, H3K27-acetylation, which is the alternative modification of the H3K27 sites marking active gene promoters and enhancers, exhibited a marked global increase, including the *PRL* and *IGFBP1* genes, indicating widespread gene activation (Grimaldi et al., 2011). Subsequent studies in essence confirmed these findings by both genome-wide and candidate gene approaches (Tamura et al., 2014, 2018; Katoh et al., 2018) and implicated another gene-activating histone modification, H3K4me3, in the transformation process. Further, insulin signaling was one of the major pathways associated with genes up-regulated by the two activating histone modifications (Tamura et al., 2014). Following up on this line of investigations, Jozaki et al. (2019) determined that decidualization was dependent on the availability of glucose for the endometrial cells. In low glucose medium acetylation of the *FOXO1* promoter regions and expression of *PRL* and *IGFBP1* were suppressed. FOXO1 is a transcription factor with pivotal involvement in decidualization (Grinius et al., 2006; Park et al., 2016). Remarkably, glucose up-regulated *FOXO1* expression and promoter H3K27 acetylation in the decidualizing endometrial stromal cells, which in turn induced *PRL* and *IGFBP1* expression associated with promoter H3K27 acetylation (Jozaki et al., 2019). Although the histone acetyl transferase(s) that function in the glucose sensing mechanism were not identified, the results further support the fundamental role of epigenetic histone modifications in decidual transformation.

At term, the decidua acquires a pro-inflammatory phenotype (Norwitz et al., 2015) that involves the emergence of myofibroblast cells (Nancy et al., 2018). Nuclear accumulation of the H3K27me3 demethylase, lysine demethylase 6A (KDM6A), has been detected concomitantly with this process, which suggests that epigenetic mechanisms, including the removal of the suppressive H3K27me3 mark, take part in the transition (Nancy et al., 2018). Very recently, a comprehensive analysis of chromatin landscape changes has been reported in endometrial fibroblasts decidualized *in vitro* (Sakabe et al., 2020). Chromatin accessibility using the ATAC-seq technique, ChIP-seq with H3K4me1, H3K27ac, and H3K4me3 antibodies, and promoter capture Hy-C to detect distant regulatory elements, were integrated and matched with RNA-seq data in this large-scale genome-wide study. In general agreement with previous findings, the results showed extensive changes during decidual transformation, particularly at enhancer sites marked with H3K4me1 and H3K27ac. Importantly, these chromatin data were also integrated with a genome-wide association study (GWAS) dataset that involved 43,568 women and explored genetic associations with gestational duration and preterm birth (Zhang et al., 2017). The computational integration of the GWAS with the functionally annotated chromatin regions in decidualized cells increased the hereditability estimates of gestational duration and resulted in the discovery of additional non-coding chromatin loci and associated genes, such as the gene encoding the transcription factor, Heart And Neural Crest Derivatives Expressed 2 (HAND2), potentially linked to gestational length (Marinić et al., 2020). From the epigenetic perspective, these studies have revealed that epigenetically controlled genomic loci involved in decidual transformation are critical for determining gestational length in women.

### Non-coding RNAs

Information about miRNAs controlling endometrial receptivity and decidualization has been reviewed previously, highlighting the Let-7, miR-200, miR-181, miR-542, and miR-30 family and miR-17-92 cluster members in these events (Liu et al., 2016, 2019). Steady expression of Let-7 miRNA isoforms has also been reported in late gestation fetal membranes/decidua (Chan et al., 2013). In decidualization models of human endometrial stromal cells *in vitro*, two microarray analyses tested 435 (Qian et al., 2009) and 1,205 (Tochigi et al., 2017) human miRNAs for differential expression. In the former study, 16 and 33 miRNAs were found to be up- and down-regulated, respectively, while in the latter study, 1 was shown up-regulated and 5 down-regulated under similar threshold criteria. There was no overlap between the two differential expression datasets. Likewise, miR-181a up-regulation was found critical for decidual transformation in one study (Zhang et al., 2015), while miR-181, -183, and -200 family members were found down-regulated in decidualized cells other investigations (Qian et al., 2009; Estella et al., 2012) reporting that reduced hsa-miR-222 expression was particularly crucial (Qian et al., 2009). Jimenez et al. (2016) described the participation of up-regulated miR-200 in decidualization *in vitro*, and potentially *in vivo*, as part of the miR-200/ZEB regulatory network (Gregory et al., 2011), which confirms that endometrial stromal cells undergo mesenchymal-to-epithelial transition during decidual transformation (Zhang et al., 2013). Furthermore, *in vitro* decidualization also appear to involve the induction of the long intergenic non-coding RNA, LINC00473 (Liang et al., 2016). Further work shall reconcile the incongruous data from different laboratories and reveal a consistent picture about the participation of particular RNA species in decidual cell differentiation and function. The importance of short and long non-coding RNAs in these processes, however, is beyond doubt.

At term labor, down-regulation of microRNAs targeting inflammatory genes has been detected in (unseparated) choriodecidua samples leaving the contribution of the maternal (decidua) and the fetal (chorion laeve) components to be determined (Montenegro et al., 2009; Stephen et al., 2015).

Finally, there is considerable interest in defining the role of decidual non-coding-RNAs in the pathogenesis of early pregnancy loss (Dong et al., 2014; Wang et al., 2015; Hong et al., 2018; Zhao et al., 2018), pre-eclampsia (Zhao et al., 2014; Lv et al., 2018; Tong et al., 2018; Moradi et al., 2019) and in the establishment of the persistent decidual phenotype after implantation (Lv et al., 2016; Wang et al., 2016). Alterations of miRNA profiles have been demonstrated under these conditions, but the detailed discussion of these aspects of miRNA involvement are beyond the scope of this review.

## Discussion

**FIGURE 1:**
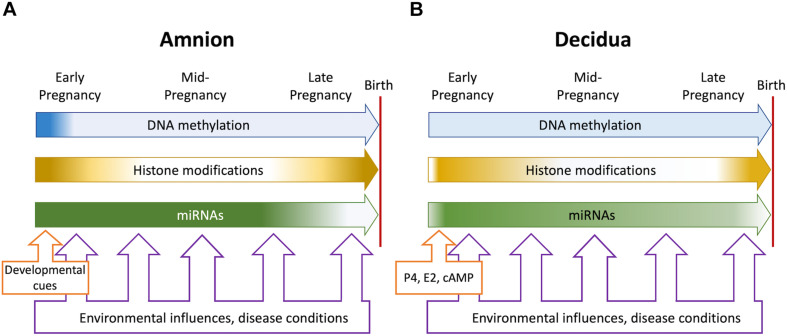
Overall dynamics of epigenetic events in the amnion **(A)** and the decidua **(B)** during pregnancy. The blue, orange, and green block arrows indicate DNA methylation, histone modifications and miRNAs, respectively. Shading denotes changing levels and shifting genome-wide distributions. In the amnion, DNA methylation and histone modification patterns are established in early gestation to support pregnancy. Histone modification patterns change at term when labor-associated inflammatory genes are activated, and tissue remodeling occurs. Micro-RNAs stabilize the protective transcriptome until term, when levels decline concomitantly with inflammatory gene activation. In the decidua, hormonal influences (progesterone, estrogens, and cAMP signaling) trigger differentiation to the pregnancy-protective phenotype. The process involves major changes in histone modifications and miRNA expression but relatively modest alterations in DNA methylation. At term, histone modifications change, and key miRNAs decline to foster a proinflammatory and labor-promoting phenotype. Environmental adversities and disease conditions may be present throughout pregnancy but most likely impact on epigenetic events during periods of dynamic change. The resulting epigenetic “footprints” may influence gene expression patterns contributing to fetal membrane disfunction and may signify fetal exposure to unfavorable intrauterine conditions.

The information currently available about epigenetic events in the fetal membranes and the decidua establishes structural changes of the chromatin as an important contributor to gene regulation in these tissues throughout pregnancy and in labor. The major types of chromatin modifications, such as DNA (CpG) methylation and histone modifications, have been found and many of them located in genome-wide and candidate gene-focused studies. Alterations in modification levels and patterns have been detected in association with gestational age, labor status and pathological conditions. Long and short non-coding RNAs (miRNAs), which represent a separate, but connected, branch of epigenetic regulation, have also been described and studied in detail. Figure 1 illustrates the overall gestational dynamics of DNA methylation, histone modifications and micro-RNA expression in the amnion and the decidua as deduced from the currently available data. The periods around implantation, early pregnancy and preparation for birth are the most active for epigenetic events to occur. Environmental and disease-associated inputs can impinge upon the fetal membranes throughout pregnancy, but epigenetic mechanisms are most likely to be influenced at these dynamic periods. Acknowledging all the advances and the increasing pace of new discoveries it remains clear that fetal membrane epigenetics is still in an early stage of development. Some critical gaps of knowledge and foreseeable future directions are indicated in the preceding sections and several more are outlined below.

• Most data about epigenetic events in the fetal membranes describe associations between chromatin modifications, non-coding RNAs and outcomes related to various physiological or pathological states of pregnancy. Associations do not prove causation; therefore, inferences about epigenetic involvement remain tentative. Evidence supporting functional roles can be generated by interventional studies that target components of the epigenetic machinery using inhibitors of chromatin modifying enzymes and specially designed epigenetic chemical probes (Wu et al., 2019). A few of these studies have been published with fetal membrane cells or tissues so far (Mitchell, 2006; Logan et al., 2010; Poljak et al., 2014) and the results highlight the need for careful consideration of off-target effects and toxicity. Other options including the genetic manipulation of chromatin modifying enzyme and/or epigenetic reader levels and activity still need to be exploited in fetal membrane research. Correlations with polymorphisms of epigenetic effector genes may provide corroborating, but still associative, evidence for epigenetic involvement in fetal membrane regulation. Polymorphisms of the DNMT3B and DNMT3L genes were found to associate with familial preterm birth and birth weight, respectively, but fetal membrane involvement (e.g., associations with pPROM) was not reported (Haggarty et al., 2013; Barisic et al., 2020).
• Fetal membrane tissues *in vivo* are subject to environmental exposures and lifestyle conditions such as smoking, diet, toxic substances and effects of social stress. Epigenetic mechanisms mediate the long term (even transgenerational) effects of these exposures and conditions as determined in animal models (Suter and Aagaard-Tillery, 2009). Furthermore, a subset of the epigenetic changes have been proposed to be of adaptive nature predicting future environmental challenges (Duncan et al., 2014). “Metastable epialleles,” which are genomic loci that function as epigenetic sensors of the environment, are critical in the process. Metastable epigenetic loci have been found in animals (Rakyan et al., 2002), for example, the *agouti* locus in mice (Dolinoy et al., 2007) and were tentatively identified in humans (Waterland et al., 2010; Dominguez-Salas et al., 2014). Smoking, nutrition and vitamin C intake significantly alter the incidence of pPROM indicating their impacts on fetal membrane function (Siega-Riz et al., 2003; Kyrklund-Blomberg et al., 2005; Nabet et al., 2007; Myhre et al., 2013), but the epigenetic aspects of these effects remain to be explored.
• Epigenetic events in the chorion leave are much less studied than in the amnion and the decidua. The chorion leave constitutes the fetal side of the maternal-fetal contact zone and a role in establishing and maintaining the maternal tolerance of the fetus is implied by its location (Kim et al., 2015). Chorion leave trophoblasts have a phenotype distinct from villous trophoblasts (e.g., chorion leave trophoblasts do not form syncytia) and possess a unique DNA methylation pattern (Eckmann-Scholz et al., 2012; Robinson and Price, 2015). Understanding the epigenetic aspects of fetal membrane function will require the characterization of chorion leave cell chromatin structure on a level with the other fetal membrane components.
• The amnion, chorion and decidua contain several types of cells, and experimental results with tissue samples represent a sum generated by heterogeneous cell mixtures that often vary in proportions. Chromatin modification patterns are cell specific, and epigenetic differences predominant in a particular cell type may be masked by the average. Analysis of isolated individual cell types or correction for cellular heterogeneity using cell-specific markers are approaches that can alleviate the problem of variable cell composition and increase the robustness of results. Importantly, the expanding suite of single cell epigenomic techniques (Clark et al., 2016) offers exciting new possibilities to analyze the composition and phenotype dynamics of fetal membrane cell populations and will very likely become a major research direction in the future.
• To identify chromatin loci where epigenetic alterations occur during pregnancy, labor and pathological conditions will require the generation and bioinformatic integration of genome-wide DNA methylation and histone modification datasets, as these modifications buttress the chromatin together and function in a combined fashion (Roadmap Epigenomics Consortium et al., 2015). Inclusion of transcriptomic, chromatin accessibility (Buenrostro et al., 2015) and chromatin conformation capture (Kempfer and Pombo, 2020) data in the integrative analyses are expected to inform about the functional impact of chromatin modification changes. These approaches are technically complex and computationally demanding, but also extremely informative, as has been demonstrated recently with decidual cells (Sakabe et al., 2020).
• Lastly, it is important to emphasize that advanced, genome-wide approaches and the more traditional analysis of individual candidate genes supplement each other and pursuing them in combination can result in optimal outcomes. Validation of key findings in genome-wide screens, and mechanistic studies in culture systems in general benefit from focusing on genes and regulatory regions pinpointed by genomic scale analyses. Emerging unexplored epigenetic mechanisms like non-cytosine DNA methylation (Ji et al., 2018) and protein mediated inheritance (Harvey et al., 2018) are exciting future directions in fetal membrane research.

## Author Contributions

TZ conceptualized the work and drafted the manuscript. JP provided intellectual input, edited the manuscript drafts, and finalized the figure designs. Both authors contributed to the article and approved the submitted version.

## Conflict of Interest

The authors declare that the research was conducted in the absence of any commercial or financial relationships that could be construed as a potential conflict of interest.
